# Allosteric Modulation of GABA_A_ Receptors by an Anilino Enaminone in an Olfactory Center of the Mouse Brain

**DOI:** 10.3390/ph7121069

**Published:** 2014-12-17

**Authors:** Thomas Heinbockel, Ze-Jun Wang, Patrice L. Jackson-Ayotunde

**Affiliations:** 1 Department of Anatomy, College of Medicine, Howard University, Washington, DC 20059, USA; E-Mail: zwang@howard.edu; 2 Department of Pharmaceutical Sciences, University of Maryland Eastern Shore, Princess Anne, MD 21853, USA; E-Mail: pljackson@umes.edu

**Keywords:** anticonvulsant, brain slice, enaminone, GABA, GABA_A_ receptor, inhibition, mitral cell, neuromodulation, olfactory bulb, positive allosteric modulator

## Abstract

In an ongoing effort to identify novel drugs that can be used as neurotherapeutic compounds, we have focused on anilino enaminones as potential anticonvulsant agents. Enaminones are organic compounds containing a conjugated system of an amine, an alkene and a ketone. Here, we review the effects of a small library of anilino enaminones on neuronal activity. Our experimental approach employs an olfactory bulb brain slice preparation using whole-cell patch-clamp recording from mitral cells in the main olfactory bulb. The main olfactory bulb is a key integrative center in the olfactory pathway. Mitral cells are the principal output neurons of the main olfactory bulb, receiving olfactory receptor neuron input at their dendrites within glomeruli, and projecting glutamatergic axons through the lateral olfactory tract to the olfactory cortex. The compounds tested are known to be effective in attenuating pentylenetetrazol (PTZ) induced convulsions in rodent models. One compound in particular, KRS-5Me-4-OCF_3_, evokes potent inhibition of mitral cell activity. Experiments aimed at understanding the cellular mechanism underlying the inhibitory effect revealed that KRS-5Me-4-OCF_3_ shifts the concentration-response curve for GABA to the left. KRS-5Me-4-OCF_3_ enhances GABA affinity and acts as a positive allosteric modulator of GABA_A_ receptors. Application of a benzodiazepine site antagonist blocks the effect of KRS-5Me-4-OCF_3_ indicating that KRS-5Me-4-OCF_3_ binds at the classical benzodiazepine site to exert its pharmacological action. This anilino enaminone KRS-5Me-4-OCF_3_ emerges as a candidate for clinical use as an anticonvulsant agent in the battle against epileptic seizures.

## 1. Enaminones in Drug Discovery and Development

Enaminones are the enamines of β-dicarbonyl compounds that comprised an amino group linked through a carbon-carbon double bond to a keto group (HN-C=C-C=O). [1]. Structure-activity relationship studies are ongoingbecause of the excellent anticonvulsant activity of previously synthesized aromatic-containing enaminones [2]. Specifically, a diverse series of anilino enaminones was recently synthesized and investigated as potential anticonvulsant compounds. These compounds are structurally unique compared to currently available antiepileptic drugs [3,4,5,6,7,8]. Furthermore, this class of enaminones has been shown to exhibit good-to-moderate protection in the traditional preclinical animal models, the subcutaneous pentylenetetrazol test (scPTZ) and the maximal electroshock seizure test. The scPTZ seizure model identifies compounds that inhibit the GABA antagonistic effects of pentylenetetrazol or raise the seizure threshold [9]. In terms of their anticonvulsant activities, anilino enaminones are comparable to some clinically used agents in animal models of seizures. In addition, the compounds have a minimal neurotoxicity side effect profile as well as a wider margin of safety than conventional antiepileptic drugs such as carbamazepine, valproate and phenytoin [10,11,12]. The enaminone system serves as the unique pharmacophoric structure of the anilino analogs. Together with their demonstrated diversity of bioactivities, these features provide an excellent opportunity for drug discovery and development. Here, we review the cellular effects of a small library of anilino enaminones on a key output neuron in a brain slice preparation containing the main olfactory bulb, which was used as an experimental platform to study enaminones.

Previous reports have explored the nucleus accumbens and hippocampus in the rat brain to study enaminone suppression of excitatory synaptic transmission and epileptiform activity [6,13]. The main olfactory bulb is anatomically different from those two brain regions and provides three advantages to test the cellular mechanisms of the effects of anilino enaminones [14,15]. Firstly, epilepsy-related proteins such as GABA_A_ receptors, sodium channels, ionotropic glutamate receptors and metabotropic glutamate receptors are expressed in the key output neuron type of the main olfactory bulb, the mitral cell. Secondly, mitral cells spontaneously generate action potentials with epilepsy-related proteins participating in the regulation of neuronal spiking. Thirdly, the excitability of mitral cells can be regulated by synaptic input. Therefore, mitral cells in an acute slice preparation of the main olfactory bulb serve as a good model for testing the bioactivity of enaminone compounds and to explore the mechanisms underlying their activity.

Anilino enaminones exhibit anticonvulsant activity *in vivo* [3,12,13]. One anilino enaminone, E139, inhibits EPSCs in the rat nucleus accumbens and hippocampus by increasing extracellular GABA concentrations [6,13], and by inhibiting tetrodotoxin-sensitive sodium currents to regulate action potential firing in neurons [16]. Other studies suggest different mechanisms as the basis for anticonvulsant activity. Benzylamino enaminones have a similar chemical structure to anilino enaminones with benzyl-substitution at the NH-moiety. Certain benzylamino enaminones exhibit anticonvulsant effects in neurons of rats and mice by suppressing glutamate-mediated excitation and action potential firing [17]. Substitutions at the NH-moiety change the target protein to which enaminones bind. Subsequently, enaminones with similar chemical structure may possess different modes of action. Our hypothesis is that the substituted site in enaminones contributes to the mode of action of these compounds. Recent work determined the mechanism of anticonvulsant action of three enaminone compounds that have non-*ortho*-substituted cyclohexenone [18]. Specifically, the effects of three anilino enaminones on neuronal activity of output neurons, mitral cells, in an olfactory bulb brain slice preparation using whole-cell patch-clamp recording has been examined. We will first review the structural and functional organization of the main olfactory bulb, before describing the modulation of mitral cell activity by anilino enaminones.

## 2. Organization of the Main Olfactory Bulb

**Figure 1:**
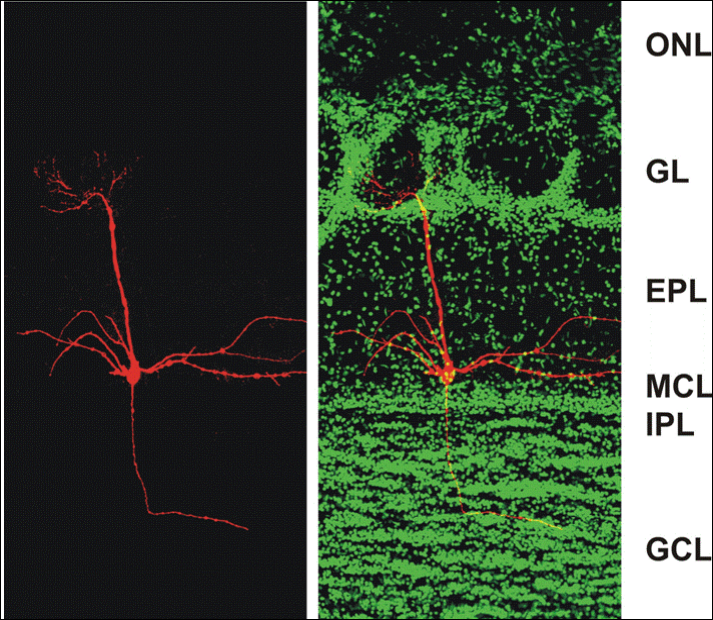
Confocal micrographs of a section of the adult mouse main olfactory bulb with a single mitral cell intracellularly filled with biocytin (**red**) and nuclei stained with Sytox Green (**green**). The mitral cell soma is located in the mitral cell layer (MCL). One apical dendrite of the mitral cell reaches into one glomerulus and several lateral dendrites span the main olfactory bulb. ONL: olfactory nerve layer; GL: glomerular layer; EPL: external plexiform layer; MCL: mitral cell layer; IPL: internal plexiform layer; GCL: granule cell layer. From [38].

The main olfactory bulb is part of the cerebral cortex, namely the allocortex rather than the neocortex, based on its cytoarchitecture and fetal development. Allocortical structures do not go through a prenatal phase of six-layered structures and instead have three or four layers in the mature brain [19]. In rodents, the main olfactory bulb is a large, layered structure that occupies almost a quarter of the length of the cranial cavity [20] and is dedicated to the processing of odorant information [14,15,21]. Input to the main olfactory bulb comes from two sources, olfactory receptor neurons in the nasal epithelium and centrifugal feedback neurons originating in different higher brain areas. Each of the bipolar olfactory receptor neurons in the nasal epithelium projects a sensory dendrite into the thin layer of nasal mucus covering the nasal epithelium, and an axon to the ipsilateral main olfactory bulb. The axon of each olfactory receptor neuron travels through the most superficial layer of the main olfactory bulb, the olfactory nerve layer, and forms synapses with neurons in one of many spheroidal neuropil structures, the olfactory glomeruli in the glomerular layer (Figure 1) (1800 glomeruli in the mouse main olfactory bulb [22], 8000 in humans [23]).

Deep to the olfactory nerve layer, the glomerular layer contains an array of olfactory glomeruli. Glomeruli are surrounded by neurons and glia [21] and house a diverse array of neuronal elements such as the axon terminals of olfactory receptor neurons. In addition, several types of local interneurons, collectively referred to as juxtaglomerular cells, send dendrites into the glomerular neuropil. Different morphological juxtaglomerular cell types can be distinguished such as external tufted cells, “short axon” cells (which actually have long axons extending through the glomerular layer [24]), and periglomerular cell [14]. Mitral/tufted cells are the main olfactory bulb output neurons. Each of them sends a single apical dendrite into one glomerulus. Olfactory receptor cells form direct synaptic contacts with mitral/tufted cells and with at least some juxtaglomerular cells [25]. Juxtaglomerular cells form synapses with many neuronal elements in the glomerular layer [26]. Periglomerular cells are GABAergic, short-axon cells whereas mitral/tufted cells are glutamatergic [27,28]. Periglomerular cells receive glutamatergic input from olfactory receptor cell axons or dendrodendritic glutamatergic input from mitral/tufted cells [14,27]. Periglomerular cells presynaptically inhibit olfactory receptor neurons through GABAergic transmission [29,30]. Mitral/tufted cells receive spontaneous bursts of inhibitory postsynaptic currents (IPSCs) from periglomerular cells at inhibitory GABAergic synapses [31,32]. In addition, centrifugal axons originating in higher olfactory centers arborize in glomeruli and may provide modulatory feedback to the glomerular layer [21,33].

The external plexiform layer lies between the glomerular and the mitral cell layer. It consists primarily of the extensive lateral dendrites of mitral/tufted cells, and granule cell dendrites sent from deeper in the main olfactory bulb. The cell bodies of mitral cells form a thin distinct band delineating the mitral cell layer, immediately below the external plexiform layer (Figure 1). Mitral/tufted cells are the principal output neurons of the main olfactory bulb. They receive olfactory receptor cell input at their apical dendrites within glomeruli, and project glutamatergic axons through the lateral olfactory tract to olfactory cortex. Each mitral/tufted cell sends a single apical dendrite into one glomerulus and several lateral dendrites (6–8) that originate at the cell body for up to thousands of micrometers through the external plexiform layer. Lateral dendrites of mitral/tufted cells form reciprocal dendrodendritic synapses with granule cell dendrites and may also receive input from centrifugal fibers and Van Gehuchten cells [34,35]. A large population (~100,000) of granule cells is found in the mitral cell layer [36], outnumbering mitral cells (~40,000) [37]. The apical dendrite of these superficial granule cells as well as the dendrite of deeper granule cells extends into the external plexiform layer to form dendrodendritic synapses with mitral/tufted cells.

The internal plexiform layer is subjacent to the mitral cell layer and contains few cell bodies but is densely packed with neuropil, including mitral/tufted cell axons and granule cell dendrites. Centrifugal axons originating in several brain regions also pass through the internal plexiform layer. The granule cell layer houses several cell types, including the prominent granule cells. These GABAergic local interneurons have no axon. Instead, they send apical dendrites to the external plexiform layer where they interact with mitral/tufted cells, and they have shorter basal dendrites receiving glutamate synapses from axon terminals (reviewed below). Little is known about the function of other neuronal types in this layer such as GABAergic short-axon interneurons, Golgi cells and Blane’s cells [21]. Blane’s cells make inhibitory synaptic contact with granule cells [39].

Immediately below the granule cell layer, in the core of the main olfactory bulb, lies an ependymal layer that is continuous with the ventricular lining in the core of the main olfactory bulb. The main olfactory bulb is a prime example for continuous neuronal generation throughout adult life such that neuronal precursors migrate to the main olfactory bulb through the rostral migratory stream. These precursors enter the granule cell layer through the ependymal layer and differentiate to become functional granule cells or they continue to the glomerular layer to differentiate into periglomerular neurons [40,41].

## 3. Processing of Sensory and Synaptic Input within the Main Olfactory Bulb

In the main olfactory bulb, glutamate is the major excitatory neurotransmitter and GABA is the main inhibitory neurotransmitter. Most synaptic processing involves serial or reciprocal glutamatergic/GABAergic circuits, principally between the dendrites of output neurons such as mitral cells and local interneurons, with specific stages of processing occurring in discrete layers of the main olfactory bulb [15]. Mitral cell activity is critical for information processing since mitral cells integrate excitatory and inhibitory synaptic input from sensory afferents, local GABAergic interneurons and centrifugal fibers originating in cortical areas.

Sensory input from olfactory receptor cells that express the same odorant receptor protein in the nasal epithelium projects to a single glomerulus and activates mitral cells innervating the glomerulus such that odor-specific patterns of input are distributed across glomeruli. One odorant-specific input channel (*i.e.*, a subset of receptor cells and their connected neurons in the main olfactory bulb such as mitral cells) influences activity in other channels through local inhibitory interneurons to enhance contrast and discriminate between odor-specific patterns of neural activity. These interneuron-mediated lateral interactions take place at two levels of synaptic processing.

Sensory input to olfactory glomeruli leads to widespread depolarization in the glomerular layer at the first level of processing. The depolarization is mediated by glutamate receptors and principally affects periglomerular cells [24]. Subsequently, these inhibitory interneurons release GABA to inhibit mitral cell responses to sensory input resulting in lateral inhibition. Short axon cells are activated by glutamate released by a population of external tufted cells instead of receiving direct glutamatergic input from olfactory afferents. Activation of short axon cells then results in widespread glutamate-mediated depolarization within the glomerular layer. Glutamate release by short axon cells activates periglomerular cells such that activation of one odorant-specific input channel can inhibit the responses of surrounding input channels with different odorant specificities similar to center-surround processing known from the retina [24].

**Figure 2:**
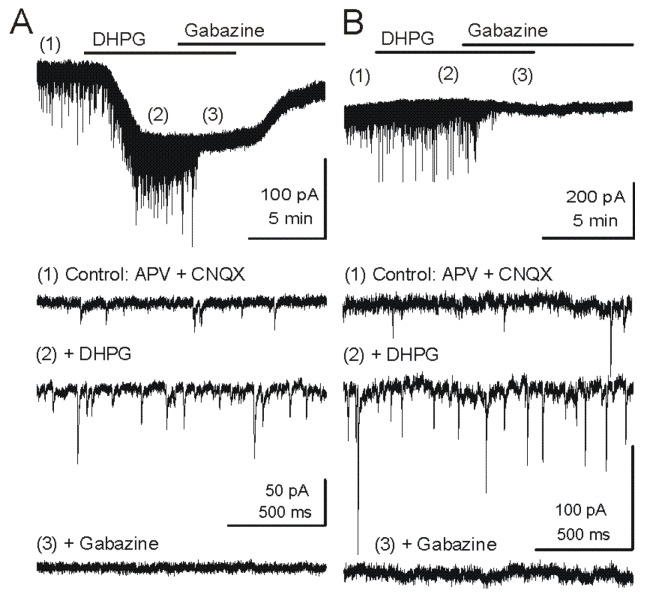
Metabotropic glutamate receptor (mGluR) group I agonist DHPG increases the frequency of GABAergic IPSCs in mitral cells. Voltage clamp recordings from MCs are made with pipettes with a high chloride concentration during blockade of iGluRs. Under these conditions, chloride-mediated IPSCs are reversed in polarity and appear as downward deflections (inward currents). (**A**) Upper trace - Application of mGluR agonist DHPG (50 μM) evokes an inward current in mitral cells recorded in slices from wildtype mice. Timepoints 1–3 are shown at faster timescale in the three lower tracers, respectively. Note the increase in frequency of fast IPSPs (trace 2), which are completely blocked by the GABA_A_ receptor antagonist gabazine (5 μM) (trace 3). (**B**) In a mitral cell from an mGluR1^−/−^ mouse, the DHPG-evoked inward current was absent, but DHPG substantially increased the frequency of IPSCs. CNQX (10 μM), APV (50 μM). Recording conditions and labeling as in (A). Modified from [44].

**Figure 3:**
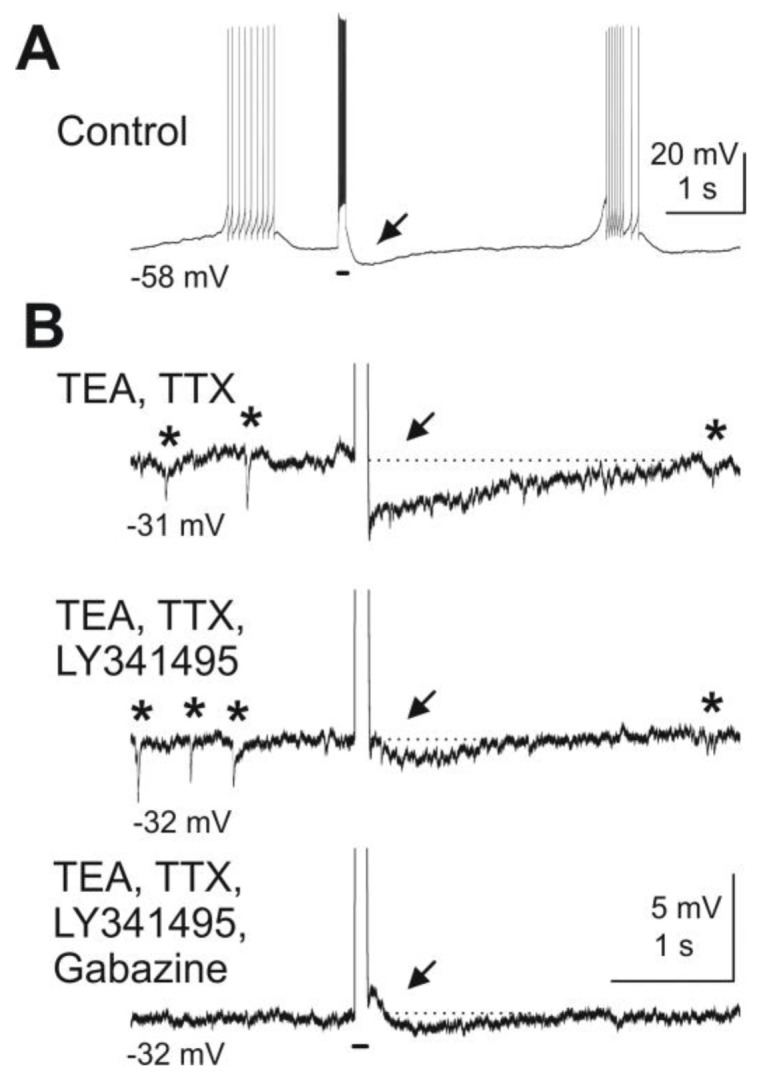
Blockade of mGluRs reduces granule cell-mediated feedback inhibition of mitral cells. Depolarizing current pulses injected into a mitral cell evoked granule cell-mediated feedback IPSPs. (**A**) A depolarizing current pulse (100 ms, 300 pA, indicated by bar beneath the trace) evoked a burst of action potentials followed by an IPSP (at arrow). (**B**) After wash-in of TEA (5 mM) and TTX (1 μM) and current clamp of the mitral cell at *ca.* −30 mV, spontaneous IPSPs are visible (marked by asterisks above the trace) and current pulses revealed a feedback IPSP (first trace). The mGluR antagonist LY341495 (100 μM) reduces the IPSP in amplitude (second trace). Addition of the GABA_A_ receptor blocker gabazine (10 μM) completely abolishes any remaining feedback IPSP as well as spontaneous IPSPs (third trace). Modified from [44].

At the second level of processing, in the external plexiform layer, mitral/tufted cell dendrites interact with dendrites of GABAergic granule cells [42]. Mitral cells release glutamate at their long lateral dendrites to activate granule cells which in turn release GABA [43] to inhibit the output of surrounding mitral cells that are part of different odorant-specific input channels. Both ionotropic and metabotropic glutamate receptors (mGluRs) facilitate feedback and feedforward inhibition of mitral cells (Figure 2 and Figure 3) [38,44,45]. Activation of mGluR5 on granule cells increases GABAergic inhibition of mitral cells. In the presence of ionotropic glutamate receptor blockers, the Group I mGluR agonist DHPG increases the frequency of spontaneous IPSCs (sIPSCs) and miniature IPSCs (mIPSCs) in mitral cells [44,46]. Mitral cells express mGluR1 and respond to DHPG with an inward current in addition to the IPSCs evoked by GABA release from granule cells.

Inactivation of mGluRs attenuates mitral cell-evoked excitation of granule cells. As previously reported [47], depolarizing current steps applied to mitral cells in the presence of sodium channel blocker TTX and potassium channel blocker TEA are followed by a feedback IPSP in mitral cells. Blockade of mGluRs reduces granule cell-mediated feedback inhibition of mitral cells (Figure 3).

In summary, the main olfactory bulb employs as a serial, two-stage, and lateral inhibitory network. The first stage is found at the level of input to mitral cells and the second stage takes place at the level of output from mitral cells and is possibly regulated by odorant-specific feedback from the olfactory cortex [24,48,49]. The purpose of this serial processing might lie in a reduction of cellular noise while enhancing contrast between odorant-specific input channels to sharpen odorant representation. Inhibitory interneurons such as periglomerular cells and granule cells act locally to provide feedback and lateral inhibition through dendrodenditic interactions with mitral cells [50].

## 4. Epilepsy and the Balance of Excitation and Inhibition

Epilepsy is a relatively frequent, long-term neurological disorder that affects 1% of the world population [51]. It is characterized by excessive temporary neuronal discharges resulting in uncontrolled convulsions referred to as epileptic seizures. Epileptic seizures can be brief (seconds) or last for a long time and are convulsive or non-convulsive. The underlying cause of epilepsy is typically unknown even though several pathological conditions can result in epilepsy such a brain injury, stroke or drug abuse. It is known that epileptic seizures result from excessive and abnormal cortical neuronal activity [52]. During an epileptic seizure, neuronal activity at a seizure focus is poorly controlled and subsequent electrical excitation spreads in brain circuits [53]. While epilepsy cannot be cured, most cases of epilepsy are treatable with medication such that patients can lead a normal life, possibly with some restrictions regarding driving a motor vehicle [54]. Despite the optimal use of available antiepileptic drugs, 25%–40% of patients are still considered to be refractory to existing drugs while others experience seizure control at the expense of acute adverse effects [55]. Therefore, discovery of novel antiepileptic agents with improved efficacy and side effect profiles is a key milestone in the battle against epilepsies. Not surprisingly, most anti-seizure medications inhibit neuronal excitability by modulating the function of several types of proteins such as sodium channels, NMDA receptors and GABA receptors [56]. Neuronal excitability in the brain is the integral of intrinsic membrane conductances and synaptic inputs. Neuronal excitability at rest is regulated by both excitatory and inhibitory inputs [57]. Mitral cells in the main olfactory bulb exhibit spontaneous action potential firing as a pattern of neuronal activity [15], which can be modulated by intrinsic membrane receptors as well as synaptic inputs [14,50]. Mitral cells express high levels of different receptors such as GABA receptors (GABA_A_, GABA_B_), ionotropic and metabotropic glutamate receptors (NMDA, AMPA, mGluR1, kainate), and serotonin receptors (5-HT_1A_, 5-HT_2A/C_). Most of these receptor proteins have been implicated in neurological disorders such as epilepsy [14,58,59,60]). Therefore, modulating any of these receptor proteins can change neuronal excitability as well as synaptic input-output relations and by extension can be critical for excitation-inhibition balance in neurons. The focus of this review is on the effects of a small library of enaminones on neuronal activity of mitral cells and the mechanisms underlying the inhibitory or excitatory actions of these enaminone compounds. The analogs are non-*ortho*-substituted cyclic enaminones and suppress neuronal excitability through activation of GABA_A_ receptors and display the characteristics of a positive allosteric modulator.

## 5. Synthesis of Anilino Enaminones

**Figure 4:**
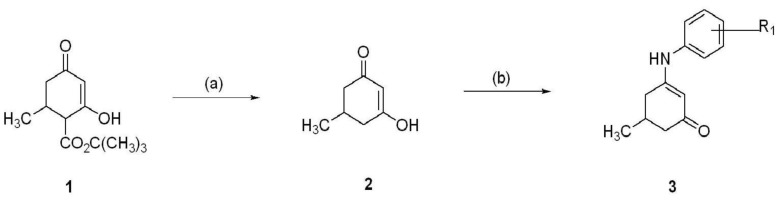
Synthesis of aniline enaminones. From [18].

**Figure 5:**
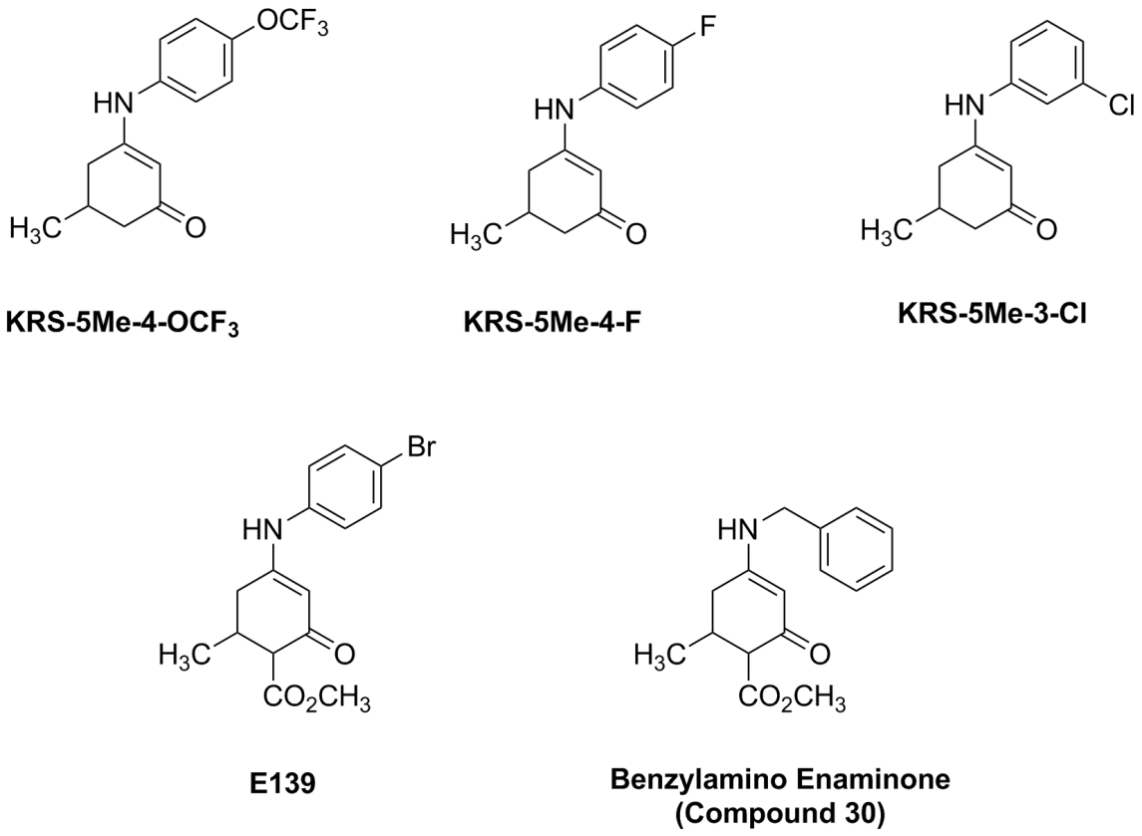
Chemical structure of aniline enaminone analogs. The difference among the three mono methyl compounds is the anilino substitution represented by *meta*-Chloro; *para*-Fluoro, and *para*-trifluoro. E139 is a *para*-bromo anilino enaminone derivative with a methyl ester substituted moiety at cyclohexenone and Compound 30 is the benzylamino analog. From [18].

The anilino enaminones reviewed here, KRS-5Me-4-OCF_3_ (5-Methyl-3-(4-trifluoromethoxy-phenylamino)-cyclohex-2-enone), KRS-5Me-4-F (3-(4-Fluoro-phenylamino)-5-methyl-cyclohex-2-enone) and KRS-5Me-3-Cl (3-(3-Chloro-phenylamino)-5-methyl-cyclohex-2-enone), were recently synthesized via a condensation reaction of the 5-methyl diketone with the corresponding substituted aniline derivatives (Figure 4). Their chemical structures are shown in Figure 5. More specifically, the mono methyl anilino enaminones (**3**) were prepared from the 5-methylcyclohexane-1,3-dione (**2**) form by the decarboxylation of 4-carbo-tert-butoxy-5-methylcyclohexane-1,3-dione (**1**), and were refluxed with appropriate substituted aniline derivatives under standard conditions [11]. The β-hydroxy keto tert-butoxy ester was prepared as previously reported [3,61,62]. The enaminone structures were confirmed via NMR analyses at 400 MHz. The analogs were subsequently evaluated for anticonvulsant activity by the National Institute of Neurological Disorders and Stroke Anticonvulsant Screening Program, National Institutes of Health. These enaminones have shown maximal electroshock seizure and scPTZ rodent activity.

*Conditions*: (a) H_2_SO_4_, Δ; (b) Δ, substituted amine.

## 6. Anilino Enaminones Inhibit Activity of Mitral Cells

**Figure 6:**
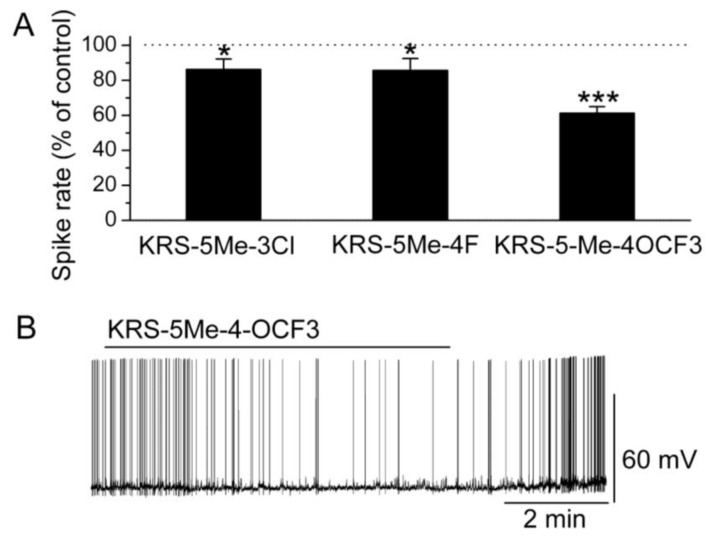
Anilino enaminones depress the spiking activity of mitral cells. (**A**) Normalized bar graph shows inhibition of spiking of mitral cells in response to bath application of KRS-5Me-4-OCF_3_ (20 μM), KRS-5Me-3Cl (20 μM), and KRS-5Me-4F (20 μM). Responses to enaminones are normalized with respect to control conditions (* *p* < 0.05, *** *p* < 0.001). (**B**) Original recording from a representative mitral cell illustrates the inhibition in firing rate and hyperpolarization following application of KRS-5Me-4-OCF_3_. From [18].

In brain slices, mitral cells spontaneously generate action potentials at 1 to 6 Hz [14]. Bath application of either KRS-5Me-3Cl or KRS-5Me-4F modulates the spike rate and membrane potential of mitral cells (Figure 6) [18]. KRS-5Me-4-OCF_3_ shows a more potent inhibition of mitral cell activity compared to the other two compounds (Figure 6A,B). KRS-5Me-4-OCF_3_ (20 μM) reversibly decreases mitral cell firing accompanied by hyperpolarization of the membrane potential. Between these three anilino enaminones, KRS-5Me-4-OCF_3_ is the most potent compound in terms of reducing spiking activity of mitral cells. Therefore, enaminone KRS-5Me-4-OCF_3_ is potentially a more potent candidate as an anticonvulsant and has been used to determine the mechanism underlying its inhibitory effect on neuronal activity [18]. Anilino enaminone KRS-5Me-4-OCF_3_ and its analogs display inhibitory effects on neuronal activity with different potencies depending on the chemical structure and concentration of the enaminone. This is consistent with previous *in vivo* results that KRS-5Me-4-OCF_3_ is the most potent anticonvulsant agent [11].

A specific substituted site in the chemical structure of enaminones may be required for receptor targeting and for conferring anticonvulsant activity. A *para*-substitution of the phenyl group with –OCF_3_ evokes the most potent suppression of neuronal activity. In contrast, a *para*-, or *meta*-substitution of the phenyl group with fluoro, chloro decreases the potency of the inhibitory activity. Possibly, a substitution in the phenyl group influences the potency of the inhibitory action of enaminones most strongly. Similarly, the significance of the substitution group has been demonstrated in benzylamino enaminones in which the unsubstituted benzylamine analog compound 30 (Figure 5) shows the most potent activity in protecting rodents from seizure onset and excitatory synaptic depression [17].

**Figure 7:**
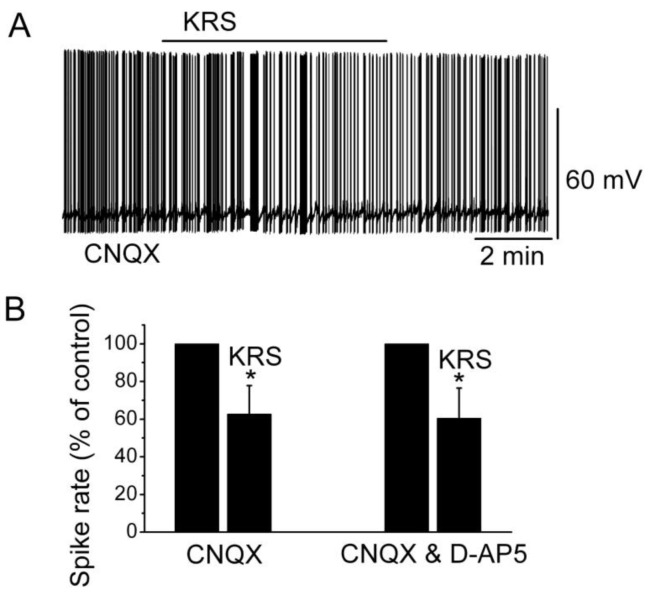
Neither AMPA/kainate nor NMDA receptor antagonists blocks the KRS-5Me-4-OCF_3_-induced inhibition of MCs. (**A**) KRS-5Me-4-OCF_3_-induced suppression of neuronal firing recorded in a representative mitral cell in the presence of CNQX (10 μM). (**B**) The normalized and averaged results showed the persistence of KRS-5Me-4-OCF_3_-induced inhibitory effects on spontaneous spiking of mitral cells in the presence of either CNQX or CNQX plus D-AP5 (50 μM) (* *p* < 0.05). From [18].

Ionotropic glutamate receptors are involved in regulating neuronal excitability in the main olfactory bulb [14]. Blockade of ionotropic glutamate receptors may result in neuronal inhibition. To determine if the inhibitory effect of KRS-5Me-4-OCF_3_ is mediated through interaction with ionotropic glutamate receptors, neuronal inhibition evoked by KRS-5Me-4-OCF_3_ can be tested in the presence of AMPA/kainate and NMDA receptor inhibitors, namely, CNQX, a potent AMPA/kainate receptor antagonist, and D-AP5, a potent NMDA receptor antagonist. In the presence of both CNQX and D-AP5, KRS-5Me-4-OCF_3_ reduces the firing rate and hyperpolarizes mitral cells not significantly different from the values of KRS-5Me-4-OCF_3_-induced suppression recorded in ACSF control condition [18]. Therefore, ionotropic glutamate receptors are not involved in the KRS-5Me-4-OCF_3_-induced MC inhibition (Figure 7).

**Figure 8:**
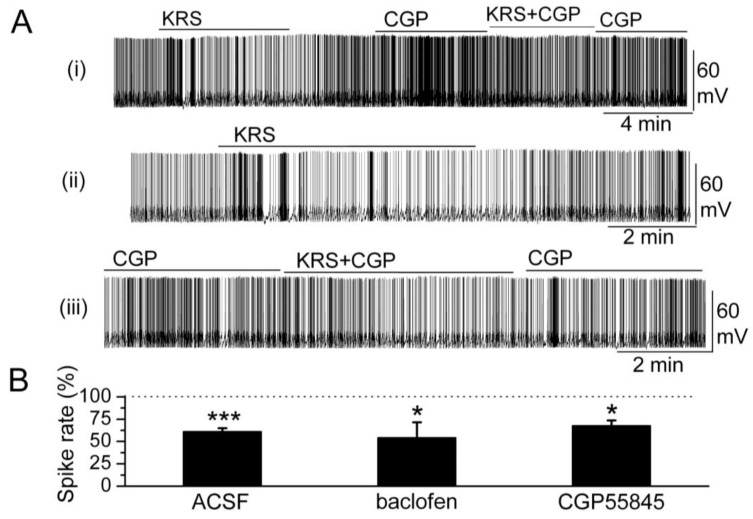
GABA_B_ receptors are not involved in KRS-5Me-4-OCF_3_-evoked inhibition. (**A**) Original recording from a mitral cell during KRS-5Me-4-OCF_3_ application in ACSF condition and in the presence of GABA_B_R antagonist CGP55845 (10 μM). The upper trace (i) illustrates the effect of KRS-5Me-4-OCF_3_ on spiking of a mitral cell and the effect of adding CGP55845. The upper trace is shown at an extended time scale in the middle (ii) and lower trace (iii). (**B**) The normalized results showed the persistence of KRS-5Me-4-OCF_3_-evoked inhibitory effects on spiking of mitral cells in the presence of the GABA_B_ receptor antagonist CGP55845 (10 µM) and the GABA_B_ receptor agonist (R)-baclofen (50 µM) (* *p* < 0.05, *** *p* < 0.001). The data for the effect of KRS-5Me-4-OCF_3_ on spiking of mitral cells were normalized with respect to ACSF, or CGP88545 or baclofen alone. From [18].

In the main olfactory bulb, GABA_B_ receptors (GABA_B_Rs) are found mainly in the glomerular layer [63,64]. GABA_B_ receptors belong to the class of metabotropic trans-membrane receptors and are linked via G-proteins to potassium channels [65]. Activation of GABA_B_ receptors can stimulate the opening of K^+^ channels to hyperpolarize the membrane potential, reduce neuronal excitability and, subsequently, prevent release of neurotransmitter. Activation of GABA_B_ receptors is known to reduce mitral cell excitability [66,67]. KRS-5Me-4-OCF_3_-evoked reduction of mitral cell firing and membrane hyperpolarization persists in the presence of GABA_B_R antagonist CGP55845 (10 μM) (Figure 8). The GABA_B_ receptor agonist (R)-baclofen (50 μM) alone evokes a strong decrease in firing and hyperpolarizes the membrane potential of mitral cells. In the presence of GABA_B_R agonist baclofen, KRS-5Me-4-OCF_3_ further reduces mitral cell firing and hyperpolarizes the membrane potential indicating that the inhibitory effect of KRS-5Me-4-OCF_3_ persists irrespective of GABA_B_ receptor activation or blockade [18].

## 7. Blockade of GABA_A_ Receptors Reverses Enaminone-Induced Inhibition of Mitral Cells

**Figure 9:**
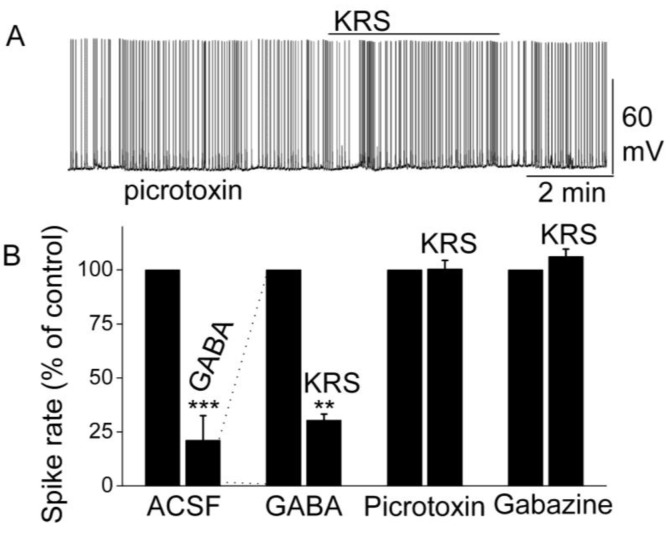
KRS-5Me-4-OCF_3_-evoked inhibition of mitral cell spiking activity is blocked by GABA_A_ receptor antagonists. (**A**) Original recording obtained from a mitral cell showed that picrotoxin (20 µM) blocks 20 µM KRS-5Me-4-OCF_3_-evoked suppression of neuronal firing. (**B**) The normalized and averaged results showed KRS-5Me-4-OCF_3_-evoked inhibitory effects on spiking of mitral cells in the presence of GABA_A_ receptor agonist GABA (50 µM), and antagonist picrotoxin (20 µM) and gabazine (5 µM) (** *p* < 0.01, *** *p* < 0.001). The data for the effect of GABA or KRS-5Me-4-OCF_3_ on spiking of MCs are normalized with respect to control condition. From [18].

Activation of GABA_A_ receptors on mitral cells suppresses their excitability and activity by decreasing the firing rate and hyperpolarizing the membrane potential [68,69]. GABA directly inhibits mitral cells; an observation that corresponds with the finding that GABA receptors are abundant in mitral cells [69,70]. In rodents, the GABA_A_ receptor has been found to form pentameric complex of unknown stoichiometry with subunits of four classes (alpha 1 to alpha 6, beta 1 to beta 3, gamma 1 to gamma 3 and delta). Specifically, mitral cells express the alpha 1, beta 1, beta 2, beta 3, and gamma 2 mRNAs strongly and the alpha 3 mRNA weakly [69]. In the presence of GABA, KRS-5Me-4-OCF_3_ reduces the firing of mitral cells even further [18]. This enhancement of the KRS-5Me-4-OCF_3_-evoked inhibition of mitral cells in the presence of increased extracellular GABA levels (50 µM) indicates that KRS-5Me-4-OCF_3_-evoked inhibition is mediated by direct activation of GABA receptors rather than by increased GABA levels (Figure 9). An increase in endogenous GABA level can result from increased GABA release or from reduced GABA re-absorption and/or degradation. Increased GABA levels or blockade of GABA_A_ receptors can result in over excitation of neurons and epileptiform activity.

The inhibition of mitral cell activity by KRS-5Me-4-OCF_3_ can be eliminated by blocking GABA_A_ receptors with receptor antagonists such as picrotoxin or gabazine (Figure 9). This result points to a KRS-5Me-4-OCF_3_ effect that is mediated either through enhancing extracellular GABA levels or a direct action on GABA_A_ receptors. The latter option is supported by the fact that, in voltage clamp recordings from mitral cells, KRS-5Me-4-OCF_3_ produces an outward current (~15 pA), which is blocked in the presence of gabazine [18].

**Figure 10:**
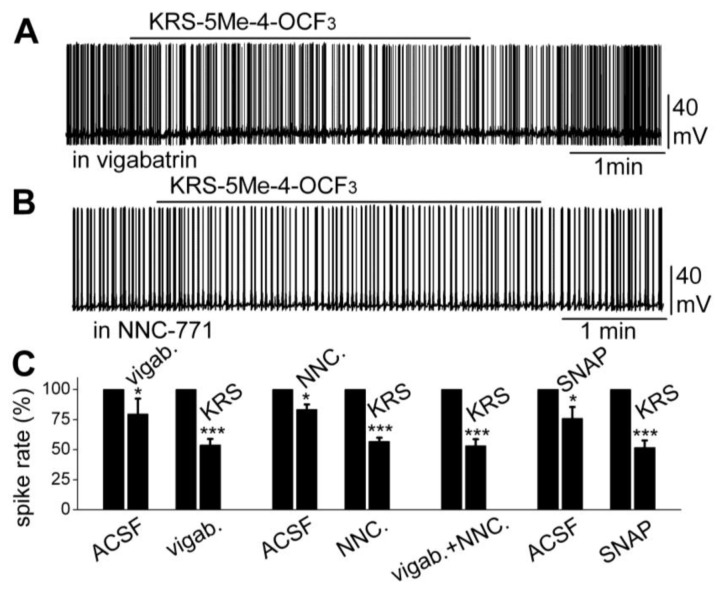
Neither an inhibitor of GABA reuptake nor an inhibitor of GABA degradation enzyme GABA-T blocked the KRS-5Me-4-OCF_3_-evoked mitral cell inhibition. (**A**) Original recording from a mitral cell showing that spiking inhibition evoked by KRS-5Me-4-OCF_3_ persisted in the presence of vigabatrin (200 µM). (**B**) Original recording from a mitral cell showing that spiking inhibition evoked by KRS-5Me-4-OCF_3_ persisted in the presence of NNC-711 (10 µM). (**C**) Summary of results from normalized and averaged data (* *p* < 0.05, *** *p* < 0.001). SNAP (20 μM). From [18].

Furthermore, increasing extracellular GABA levels does not block the KRS-5ME-4-OCF_3_-induced inhibition of neuronal excitability. The fate of extracellular GABA can be described by two scenarios [71]; in one scenario, extracellular GABA is enzymatically degraded; and alternatively, after GABA is released from synaptic terminals, it may be removed from the extracellular space by GABA uptake back into axon terminals and/or into glial cells by plasma membrane transporters (GATs). Captured GABA is then degraded by the enzyme GABA transaminase (GABA-T). Vigabatrin is an irreversible and selective GABA-T inhibitor that results in extracellular accumulation of GABA in the synaptic cleft. It induces a reduction in mitral cell firing rate. In the presence of vigabatrin, application of KRS-5Me-4-OCF_3_ results in further reduction of the mitral cell-firing rate accompanied by hyperpolarization of the membrane potential (Figure 10A,C). The persistence of the KRS-5Me-4-OCF_3_ effect in the presence of vigabatrin indicates that enaminone-evoked neuronal inhibition is not mediated by GABA-T [18].

The next questions to answer were if enaminones interact with GATs to regulate GABA re-uptake and if GATs modulate KRS-5Me-4-OCF_3_-evoked neuronal inhibition. NNC-711 is a potent and selective inhibitor of GABA uptake by GAT-1 and (*S*)-SNAP 5114 is a selective inhibitor by GAT-2 and GAT-3. Bath application of either NNC-711 or (*S*)-SNAP 5114 reduces mitral cell spiking and modulates the membrane potential. In the presence of either GABA-uptake blocker, KRS-5Me-4-OCF_3_ further reduces the firing rate and hyperpolarizes mitral cells (Figure 10B,C). Even in the presence of both the GABA reuptake inhibitor NNC-711 and the GABA-T inhibitor vigabatrin, the inhibitory effect of KRS-5Me-4-OCF_3_ remains fully intact (Figure 10C). Neither the GABA reuptake transporters, GATs, nor GABA transaminase, GABA-T, interfere with KRS-5Me-4-OCF_3_-evoked neuronal inhibition, indicating that an extracellular increase of GABA does not contribute to the pharmacological effects of KRS-5ME-4-OCF_3_ [18].

Both GABA_A_ and GABA_B_ receptors are present in the main olfactory bulb but participate in the regulation of mitral cell excitability in distinct ways. Whereas GABA_A_ receptors directly regulate the excitability of mitral cells, GABA_B_ receptors indirectly regulate of mitral cells via presynaptic inhibition of olfactory receptor neuron terminals [14,50]. Anti-epileptic drugs such as benzodiazepines are known to interact with GABA_A_ receptors [53], which confirms the observation that KRS-5Me-4-OCF_3_ does not interact with GABA_B_ receptors but rather interacts directly with GABA_A_ receptors to decrease neuronal activity of mitral cells.

The chemical structure of enaminones is critical in determining their mode of action in terms of neuronal inhibition and anticonvulsant effects. E139 (Figure 5) suppresses excitatory synaptic transmission by enhancing extracellular GABA levels [6] and blocking TTX-sensitive sodium channels and, thereby, directly inhibiting postsynaptic neuronal excitability [16]. Other enaminones with chemical moieties different from KRS-5Me-4-OCF_3_ interact with GABA_A_ receptors [72,73,74]. The comparative molecular field analysis (CoMFA) and comparative molecular similarity (CoMSIA) techniques provide additional enaminone derivatives that most likely target GABA receptors. These techniques generate models to define the specific structural and electrostatic features essential for enhanced binding of enaminones to the putative GABA receptor [75].

## 8. A Substituted Anilino Enaminone Exhibits Characteristics of a Positive Allosteric Modulator of GABA_A_ Receptors

**Figure 11:**
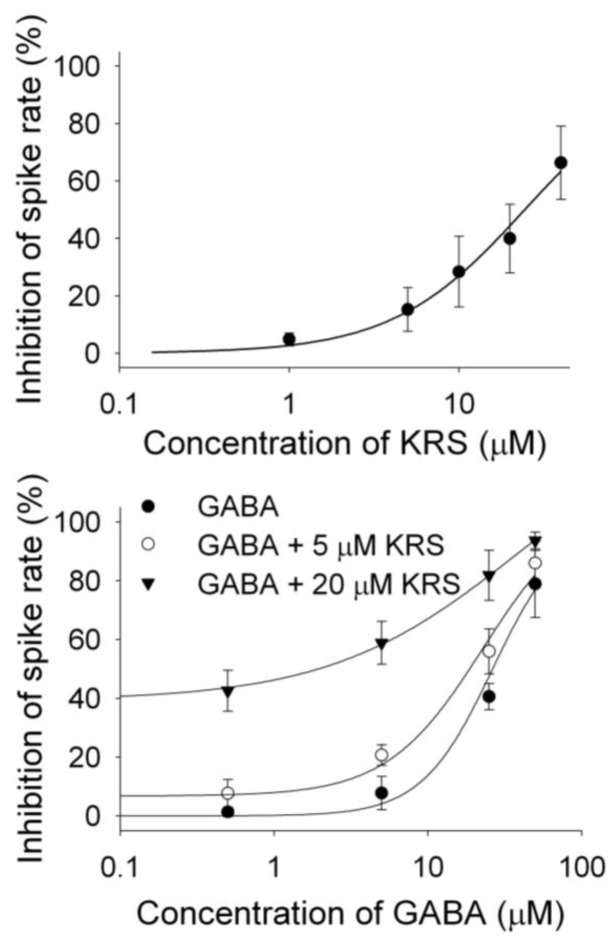
The concentration–response curves of KRS-5Me-4-OCF_3_–evoked inhibition and of GABA in the presence of KRS-5Me-4-OCF_3_. (**A**) The KRS-5Me-4-OCF_3_–evoked change in spiking rate was normalized to the control condition, and then averaged. Each point was the mean value ° SEM of 4 to 7 cells. The line is fit for the data to the Hill equation: *y =* *Y_0_* + *Ax^n^*/(*K_d_^n^ + x^n^*), where *y* is the inhibition of spiking rate, *Y_0_* is minimal inhibition, *A* is maximal inhibition, *K_d_* is the apparent dissociation constant for agents, and *n* is the Hill coefficient. *K_d_* and *n* were estimated using a Marquadt nonlinear least-squares routine. (**B**) Shift of the concentration–response curves of GABA in the presence of KRS-5Me-4-OCF_3_ at different concentrations (0, 5, 20 μM). The lines are fits for the data to the above Hill equation. From [18].

KRS-5Me-4-OCF_3_ enhances the inhibitory effect of GABA on mitral cells (Figure 9B), suggesting that KRS-5Me-4-OCF_3_ acts as a positive allosteric modulator of GABA_A_ receptors. GABA_A_ receptors are the main target for positive allosteric modulators such as benzodiazepines [76,77,78]. A positive allosteric modulator facilitates GABA_A_ receptor function by binding at a site distinct from the GABA binding site and by increasing GABA affinity for the GABA_A_ receptor. This can be tested by examining the concentration-response relationships of KRS-5Me-4-OCF_3_ and GABA in the absence and presence of KRS-5Me-4-OCF_3_ (0 μM, 5 μM, 20 μM) (Figure 11) [18]. The stoichiometry of drug and receptor interaction is 1:1 based on a fitted Hill coefficient (*n*) value of 1.11. The inhibitory effect of KRS-5Me-4-OCF_3_ is concentration-dependent with an EC_50_ of 24.5 μM. GABA-evoked inhibition is concentration-dependent (Figure 11B). The concentration-response curves are left shifted by KRS-5Me-4-OCF_3_, which suggests that KRS-5Me-4-OCF_3_ mostly likely binds at non-GABA binding sites on the GABA receptor [18]. The EC_50_ of GABA fitted by the Hill equation is 28.8 μM for GABA only, 19.9 μM for GABA plus 5 μM KRS-5Me-4-OCF_3_, and 10.5 μM for GABA plus 20 μM KRS-5Me-4-OCF_3_. KRS-5Me-4-OCF_3_ enhances the affinity of GABA for GABA binding sites suggesting an action of KRS-5Me-4-OCF_3_ as a positive allosteric modulator of the GABA_A_ receptor. The *in vivo* data, which show an anticonvulsant effect of KRS-5Me-4-OCF_3_, correspond well with the suggested cellular mechanism of its action. The corresponding *in vitro* and *in vivo* results also indicate that recording in mitral cells is an appropriate method to elucidate the bioactivity of enaminones.

## 9. KRS-5Me-4-OCF_3_ Binds at the Benzodiazepine Site

GABA is the major inhibitory neurotransmitter in the brain. The GABA_A_ receptor is a ligand-gated ion channel that binds GABA but it possesses distinct binding sites for GABA, benzodiazepines, barbiturates, ethanol [79], inhaled anesthetics, and neuroactive steroids. Compounds such as benzodiazepines, neuroactive steroids, and barbiturates act as allosteric modulators of GABA_A_ receptors and have also been identified as useful anxiolytics, anticonvulsants, anesthetics, and sedative-hypnotics. Positive allosteric modulators increase the affinity of GABA for the binding site.

*In vivo* data shows potent anticonvulsant effects of enaminones in chemically-induced epilepsy in animal models but with fewer side-effects [11,12]. The *in vivo* and *in vitro* results suggest that KRS-5Me-4-OCF_3_ binds to benzodiazepine sites on GABA_A_ receptors to exert its effect. Indeed, flumazenil, a benzodiazepine site antagonist, slightly increases mitral cell firing. However, in the presence of flumazenil, KRS-5Me-4-OCF_3_ fails to inhibit firing or change the membrane potential of mitral cells [18]. This establishes that the enhancement of GABA by KRS-5Me-4-OCF_3_ is mediated at the classical benzodiazepine site.

The enaminone KRS-5Me-4-OCF_3_ acts as a novel positive allosteric modulator to decrease neuronal activity via direct regulation of GABA_A_ receptors, which suggests that KRS-5Me-4-OCF_3_ could be a potential medication as anxiolytic, anticonvulsant, anesthetic, and sedative-hypnotic. A number of enaminone compounds provide protection against glutamate-mediated excitatory synaptic transmission by modulating GABAergic transmission [6,13]. We hypothesize the existence of an essential pharmacophore within the enaminone structure that possibly interacts with the GABA receptor. The exact site and structural requirements for optimal binding are unknown. Nevertheless, we hypothesize, because of the molecular similarities between the enaminone analogs discussed here, that they share a common binding pocket on the GABA receptor, which explains the probability of eliciting similar biological responses.

Enaminones with different chemical structure can use different mechanisms to inhibit neuronal activity. Anilino enaminone E139 suppresses excitatory synaptic transmission through increasing GABA levels [6]. Chemically, E139 differs in its structure from KRS-5Me-4-OCF3 in the C1 position of the cyclohexenone moiety. E139 has a methyl ester substitution of the cyclohexenone moiety, whereas the three compounds described here are non-esters and *para*- or *meta*-substituted in the aromatic moiety. Enaminone compounds such as KRS-5Me-4-OCF_3_ act as a positive allosteric modulator of GABA_A_ receptors. We postulate that the *para*-substitution of an aniline enaminone is a critical factor for the interaction with target proteins. Benzylamino enaminones with a similar structure to anilino enaminones use a distinctly different cellular mechanism for anti-convulsion and to suppress glutamate-mediated excitatory synaptic transmission as shown for benzylamino enaminone compound 30 [17]. Structurally diverse enaminones with different site substitutions may form different pharmacophores in order to interact with specific protein targets. A better understanding of the structure-response relationship may pave the way to rational drug design for potential anticonvulsant and anxiolytic compounds.
